# Is There a Role for Unstimulated Thyroglobulin Velocity in Predicting Recurrence in Papillary Thyroid Carcinoma Patients with Detectable Thyroglobulin after Radioiodine Ablation?

**DOI:** 10.1245/s10434-012-2391-6

**Published:** 2012-05-11

**Authors:** Hilda Wong, Kai P. Wong, Thomas Yau, Vikki Tang, Roland Leung, Joanne Chiu, Brian Hung-Hin Lang

**Affiliations:** 1 Department of Medicine, Division of Hematology and Oncology, Queen Mary Hospital, The University of Hong Kong, Hong Kong SAR, China; 2 Department of Surgery, Division of Endocrine Surgery, Queen Mary Hospital, The University of Hong Kong, Hong Kong SAR, China

## Abstract

**Background:**

In the follow-up of papillary thyroid cancer (PTC) patients treated with curative thyroidectomy and radioiodine ablation, raised thyroglobulin (Tg) predicts recurrence with reasonable sensitivity and specificity. However, a proportion of patients present with raised Tg level but no other clinical evidence of disease. Only limited data on Tg kinetics have been reported to date. Here we aim to evaluate the prognostic and predictive significance of nonstimulated serum Tg velocity (TgV).

**Methods:**

Consecutive PTC patients treated with curative thyroidectomy and radioiodine ablation between 2003 and 2010 were analyzed. Patients with at least one detectable Tg measurement (>0.2 ng/mL) were included. TgV was defined as the annualized rate of Tg change. Logistic regression analyses were performed to evaluate the role of TgV in the prediction of disease recurrence. The optimal TgV cutoff was assigned by receiver–operating characteristic curve analysis. Overall survival of patients above versus below the TgV cutoff were determined by the Kaplan–Meier method and compared.

**Results:**

Of a total of 501 patients, 87 had at least one Tg value >0.2 ng/mL; in these latter patients, 29 (33.3 %) developed recurrence. TgV was an independent predictor of the recurrence. TgV ≥0.3 ng/mL per year predicted recurrence with a sensitivity of 83.3 % and specificity of 94.4 %. Patients with TgV below the cutoff had a significantly better overall survival (*p* = 0.038).

**Conclusions:**

TgV predicts recurrence with high sensitivity and specificity, and is a prognosticator of survival in postthyroidectomy and postablation PTC patients with raised Tg.

**Electronic supplementary material:**

The online version of this article (doi:10.1245/s10434-012-2391-6) contains supplementary material, which is available to authorized users.

In papillary thyroid cancer (PTC), thyroidectomy, radioiodine (^131^I) therapy, and thyroid hormone suppression are the mainstay of treatment, conferring excellent overall survival.^1^ Despite effective initial treatment, the prognosis is significantly affected by tumor recurrence, which occurs in up to 30 % of patients at 30 years.^2^ Improvement of current methods to detect recurrence early and accurately is therefore clinically important.

Thyroglobulin (Tg) is a glycoprotein specific to differentiated thyroid tissue; after thyroidectomy and radioiodine remnant ablation, an elevated serum Tg level is a sensitive marker of residual cancer.^3^ Periodical measurement of Tg after initial thyroid ablative therapy is recommended in the monitoring of PTC patients.^4^ Nevertheless, Tg level may be affected by the various laboratory assays used.^5^ Interference may also occur in the presence of anti-Tg antibodies or heterophile antibodies.^6^^,^ 7

The sensitivity of Tg can be increased with thyroid-stimulating hormone (TSH) stimulated by either thyroid hormone withdrawal or recombinant human TSH administration.^8^^,^ 9 On the other hand, some patients may present with elevated interference-corrected Tg levels alone, without clinical or radiological evidence of disease at physical examination, neck ultrasound, diagnostic radioiodine whole body scan (WBS), and/or computed tomography (CT) or ^18^F-fluorodeoxyglucose–positron emission tomography (FDG-PET). Although possibly nonspecific especially with the development of increasingly sensitive Tg assays, this condition is generally taken to represent occult malignant disease.^10^^,^ 11 Empirical ^131^I treatment is generally recommended at a Tg level of 5 ng/mL during or 10 ng/mL off thyroxine treatment.^4^^,^ 12 However, its clinical significance and management options are in fact controversial based on the current literature. Empirical radioiodine treatment has not been demonstrated prospectively to be associated with improved outcome, while in a retrospective study the majority of patients with positive Tg and negative WBS remained free of disease at 8 years’ follow-up.^13^ Moreover, it has been postulated that benign radioresistant ectopic thyroid or thymus tissue may instead be the source of Tg secretion.^3^

In these patients, monitoring the Tg trend plays a particular role, but only limited evidence on the prognostic and predictive significance of postablation Tg kinetics has been reported to date. In the present study, we aimed to evaluate the rate of change of nonstimulated serum Tg, or Tg velocity (denoted by TgV), to predict recurrence in postthyroidectomy and postablation PTC patients who have raised Tg levels.

## Materials and Methods

### Patients

Consecutive PTC patients undergoing primary thyroidectomy with a curative intent in a tertiary referral center between 2003 and 2010 were analyzed. Owing to the adoption of different and less sensitive biochemical assays before 2003, patients who presented before this date were excluded. Patients with pathologies other than PTC, including follicular carcinoma, Hürthle cell carcinoma, medullary carcinoma, anaplastic carcinoma, and lymphoma, were also excluded. All patients underwent total thyroidectomy and neck dissection as required, with postoperative radioiodine of 3 GBq administered to ablate thyroid remnant. External-beam radiotherapy was considered in patients aged 45 or older with extrathyroidal tumor extension, according to current guidelines.^4^ Postablation WBS was performed subsequently; patients with residual or metastatic disease were further excluded.

According to specified protocols as previously described, nonstimulated serum Tg levels, TSH and free T4 were measured and physical examination performed 3 months after surgery and thereafter at each follow-up visit every 6 months, more frequently in patients with suspected relapse.^14^ Tg values with a simultaneous raised TSH level were excluded. Patients with at least one detectable Tg measurement (>0.2 ng/mL) were included in the present study; they were followed up for clinical outcomes of recurrence and survival. Patients were included irrespective of their anti-Tg antibodies, as Tg was evaluated longitudinally rather than on the basis of individual measurements. Ultrasonography of the neck was performed once every 6–12 months and chest X-ray annually, while other imaging studies including WBS, CT, or FDG-PET were performed upon clinical indication. In this study, recurrence was defined as the first relapse of disease detected on imaging, and/or histological proof where clinically feasible.

### Biochemical Assays

Tg and TSH was determined by Immunite immunoassay (Siemens Healthcare Diagnostics) and Advia Centaur TSH-3 ultra chemiluminescent immunoassay (Siemens Healthcare Diagnostics), with sensitivities of 0.2 ng/mL and 0.03 mIU/L, respectively. Anti-Tg antibodies were detected by Quanta Lite enzyme-linked immunosorbent assay (Inova Diagnostics).

### Definition of TgV

The first Tg value of >0.2 ng/mL noted during the course of follow-up was denoted by Tg1, and the two consecutive Tg values immediately after the first raised value by Tg2 and Tg3. TgV was defined as the annualized rate of Tg change. Two sets of TgV were determined in the current study. Primary TgV (TgVp) was calculated by [(Tg2 − Tg1)/(time elapsed in years)]. In addition, cumulative TgV (TgVc) was determined by the summation of [(Tg2 − Tg1)/(time elapsed in years)] and [(Tg3 − Tg2)/(time elapsed in years)], to investigate any improvement in sensitivity and specificity when the further trend of Tg change was taken into account.

### Statistical Analyses

Univariate and multivariate logistic regression analyses were performed to evaluate the role of TgV among other independent factors, including age, gender, primary tumor and regional lymph node stage, and extrathyroid invasion, in the prediction of disease recurrence. Moreover, the optimal TgV cutoff for clinical purpose was determined by receiver–operating characteristics (ROC) curve analysis. Recurrence-free survival and overall survival of patients above versus below the TgV cutoff were determined by the Kaplan–Meier method and compared by the log-rank test. Statistical analyses were performed by the statistical software SPSS Statistics, version 19. Significance was assumed at *p* < 0.05.

## Results

### Patient Demographics

**Fig. 1:**
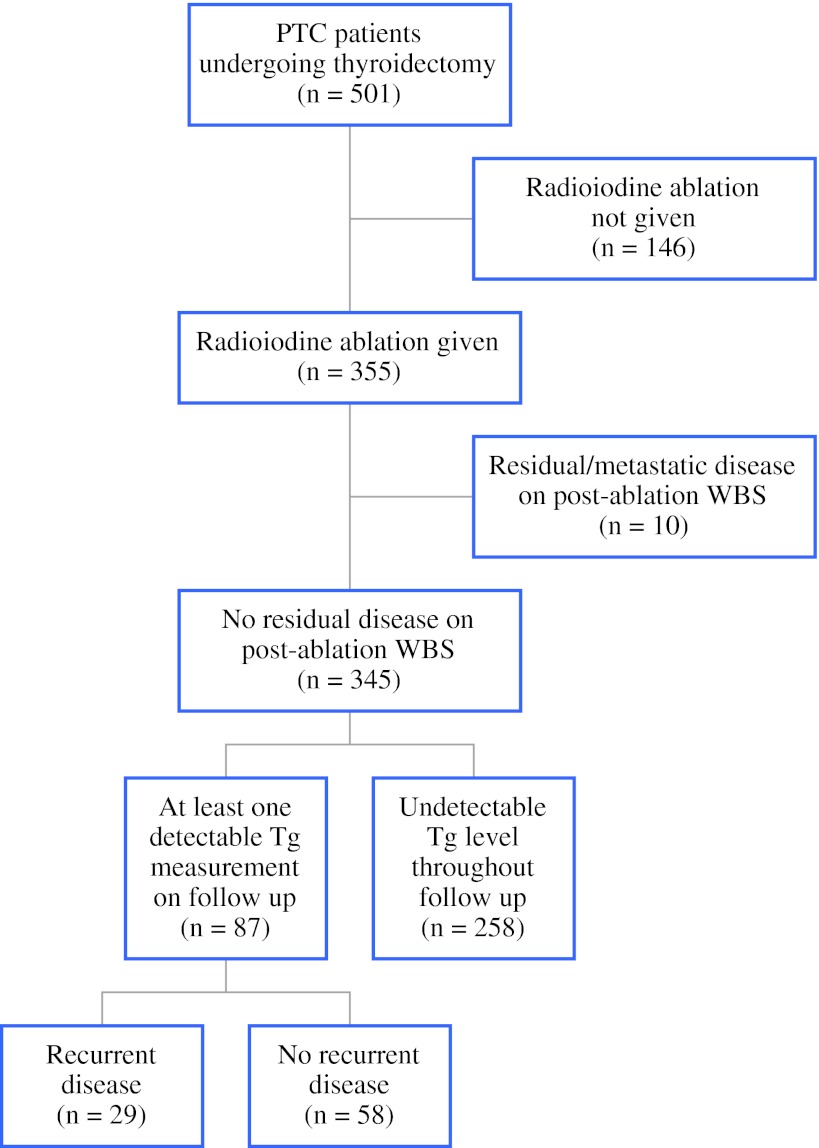
Patients undergoing thyroidectomy, radioiodine ablation, and subsequent follow-up

A total of 501 PTC patients underwent curative surgery during the study period (Fig. 1). As a result of lack of indications, presence of contraindications, or patient preference, 146 patients (29.1 %) did not receive postoperative radioiodine ablation, while 10 (0.02 %) were found to have residual or metastatic disease on postablation WBS; these patients were excluded from further analysis. Tg kinetics were evaluated in the remaining 345 patients, among whom 258 had undetectable Tg levels throughout the course of their follow-up, while 87 had at least one Tg measurement of >0.2 ng/mL. Of these latter patients, the median interval between surgery and first raised Tg values was 13.3 months; 29 (33.3 %) developed recurrence, whereas 58 (66.7 %) showed no clinical or radiological evidence of disease. The demographics of these patients are shown in Table 1.

**Table 1:** Demographics of patients with raised Tg levels after operation and radioiodine ablation treatment^a^

Characteristic	Recurrent disease	No disease	*p* value
Age, median (range)	54 (16–84)	47 (20–85)	0.320^b^
<45 years	10 (34.5)	26 (44.8)	
≥45 years	19 (65.5)	32 (55.2)	
Gender			0.321
Male	10 (34.5)	14 (24.1)	
Female	19 (65.5)	44 (75.9)	
Neck dissection			0.219^c^
Not performed	6 (20.7)	20 (34.5)	
Performed	21 (72.4)	35 (60.3)	
Unilateral	16 (55.2)	15 (25.9)	
Bilateral	3 (10.3)	5 (8.6)	
Central	2 (6.9)	15 (25.9)	
Postoperative external-beam radiotherapy			0.077^c^
Not provided	23 (79.3)	54 (93.1)	
Provided	6 (20.7)	4 (6.9)	
Primary tumor (T) stage			0.003^c^
T1–2	3 (10.3)	28 (48.3)	
T3–4	23 (79.3)	27 (46.6)	
Regional node (N) stage			0.006^c^
N0–1a	8 (27.6)	29 (50.0)	
N1b	15 (51.7)	17 (29.3)	
MACIS score, median (range)	5.97 (3.70–11.00)	5.05 (1.45–7.75)	0.011^b^
<6	13 (44.8)	36 (62.1)	
6–6.99	3 (10.3)	8 (13.8)	
7–7.99	4 (13.8)	10 (17.2)	
≥8	5 (17.2)	0 (0)	
Total no. of patients	29	58	

*MACIS* metastases, age, completeness of surgery, invasion, and size

^a^Data are presented as median (range) or *n* (%)

^b^Mann-Whitney *U* test

^c^Fisher’s exact test

In the recurrent and disease-free patients, the median ages at diagnosis were, respectively, 54 and 47 years; most patients were female in both groups, consistent with local epidemiology. Neck dissection and postoperative external-beam radiotherapy were more frequently performed in the recurrent group, although the difference did not reach statistical significance. In these patients, however, more often the primary tumor was 4 cm or larger or invaded beyond the thyroid (i.e., stages T3–4 according to the American Joint Committee on Cancer tumor, node, metastasis staging system, 6th edition), and lymph node staging was more advanced (*p* = 0.003 and 0.006, respectively).^15^ Correspondingly, the MACIS (metastases, age, completeness of surgery, invasion, and size) score was significantly higher (*p* = 0.011).^16^

In the recurrent group, 18 patients (62.1 %) had disease in the neck, 1 (3.4 %) in the tumor bed, and 7 (24.1 %) in distant sites as the first site or sites of recurrence. As treatment of recurrent disease, 18 patients (62.1 %) underwent surgical resection, 23 (79.3 %) received further radioiodine, 2 (6.9 %) received external-beam radiotherapy, and 1 (3.4 %) was treated conservatively.

### Univariate and Multivariate Logistic Regression Analyses

On univariate analysis, classical clinicopathological variables, including the presence of extrathyroidal extension (*p* = 0.006), higher primary tumor stage T3–4 vs. T1–2 (*p* = 0.001), regional lymph node stage N2 vs. N0–1a (*p* = 0.002), and higher TgV (*p* < 0.0005), but not age (*p* = 0.109) or gender (*p* = 0.157), were significantly associated with recurrence. Multivariate logistic regression produced a model, as presented in Table 2. However, only TgV remained as an independent predictor of the recurrence outcome (odds ratio 1.140, 95 % confidence interval 1.024–1.269, *p* = 0.017).

**Table 2:** Logistic regression model of various independent variables in the prediction of recurrence

Variable	B	*p* value	OR	95 % CI for OR	
				Upper	Lower
TgVp	0.131	0.017	1.140	1.024	1.269
Extrathyroid invasion	0.668	0.508	1.950	0.270	14.085
Primary tumor stage T3–4	0.573	0.602	1.773	0.206	15.258
Regional node stage N1b	0.784	0.279	2.190	0.530	9.046

*OR* odds ratio, *CI* confidence interval

### TgV Kinetics

The median time between Tg1 and Tg2 measurement and between Tg2 and Tg3 measurement were, respectively, 5.7 months (range 1.0–28.8 months) and 6.3 months (range 1.3–19.9 months). Baseline Tg1 was significantly higher in the recurrent compared to disease-free patients, while confounding TSH levels were similar (Table 3).

**Table 3:** Comparison of baseline Tg1, simultaneous TSH values, and TgVp in patients with and without recurrent disease^a^

Characteristic	Recurrent disease	No recurrence	*p* value^b^
Tg1	7.25 (0.3 to 4800.0)	1.25 (0.3 to 117.0)	<0.005
Simultaneous TSH			
With Tg1	0.03 (0.03 to 3.70)	0.06 (0.03 to 5.00)	0.064
With Tg2	0.03 (0.03 to 4.00)	0.03 (0.03 to 3.50)	0.810
With Tg3	0.03 (0.03 to 2.00)	0.03 (0.03 to 4.00)	0.283
TgVp	16.03 (−29.20 to 5383.75)	−0.67 (−27.31 to 6.80)	<0.005
Total no. of patients	29	58	

^a^Data are presented as median (range)

^b^Mann–Whitney *U* test

**Fig. 2:**
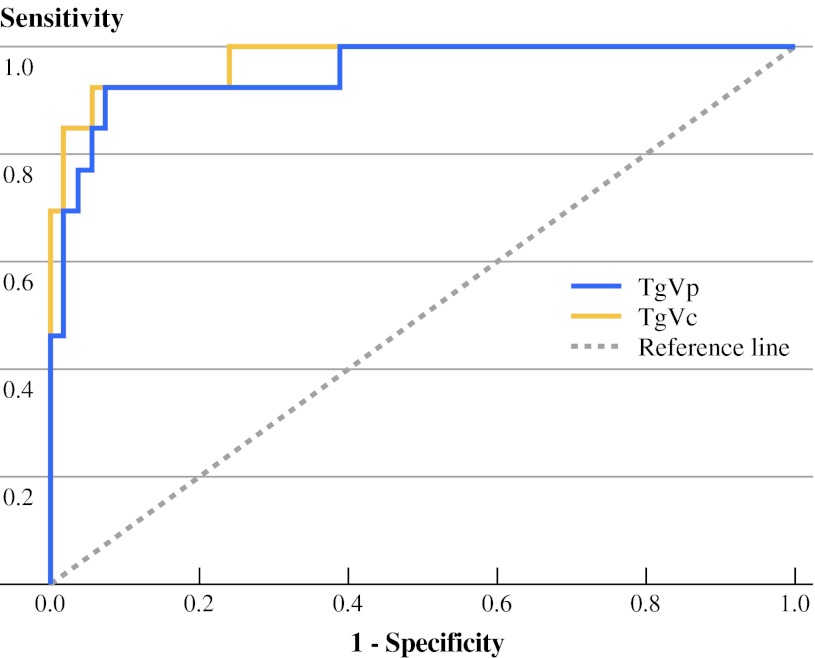
Receiver–operating characteristics (ROC) curve of primary TgV (TgVp) and cumulative TgV (TgVc)

TgVp (primary TgV, defined by [(Tg2 − Tg1)/(time elapsed in years)]) was higher in the recurrent group (Table 3). According to ROC analysis (Fig. 2), TgVp equal to or greater than a cutoff value of 0.3 ng/mL per year was found to predict recurrence with a sensitivity of 83.3 % and a specificity of 94.4 %. The sensitivity of TgV could be improved by determining TgVc (cumulative TgV, summation of [(Tg2 − Tg1)/(time elapsed in years)] and [(Tg3 − Tg2)/(time elapsed in years)]). TgVc ≥0.6 mg/mL per year predicted recurrence with a higher sensitivity of 92.3 %, while specificity remained similar at 94.4 %.

### Survival Outcomes and Recurrence Patterns

**Fig. 3:**
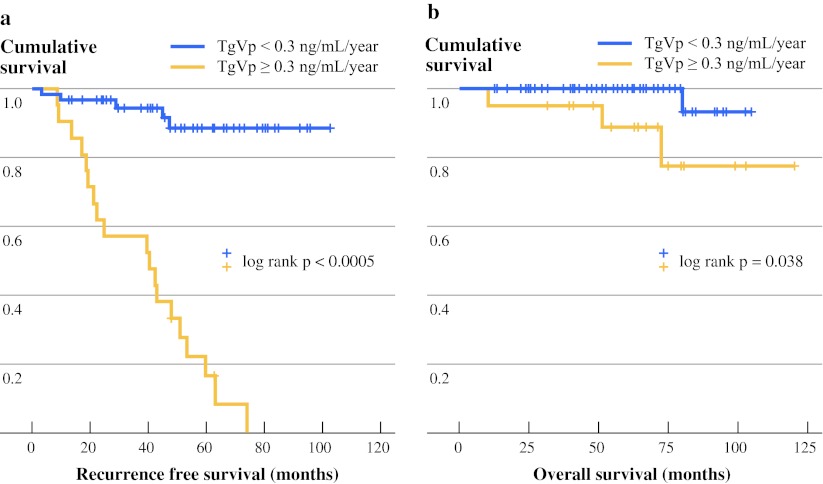
Kaplan–Meier survival curves according to TgVp cutoff of 0.3 ng/mL per year. **a** Recurrence-free survival. **b** Overall survival

At a median follow-up of 56.8 months, the median survival has not been reached. The Kaplan–Meier survival curves are shown in Fig. 3. Preliminarily, patients with TgVp <0.3 ng/mL per year had a significantly better overall survival than those with TgVp at or above the cutoff value (*p* = 0.038). In addition, all distant recurrence occurred in patients with TgVp above the cutoff value.

## Discussion

To our knowledge, the current study is the first in the literature to report the specific cutoff of TgV at 0.3 ng/mL per year to predict recurrence, especially distant relapse, in postoperative postablation PTC patients. More importantly, we have demonstrated the prognostic role of such cutoff to predict significantly different recurrence-free and overall survival.

The development of increasingly sensitive Tg assays has improved the rate of disease detection while patients are maintained on thyroxine therapy; it has been suggested that these assays reduce the need for TSH stimulation.^17^ On the other hand, test specificity may be compromised, leading to more false-positive results. While receiving thyroxine, Tg assays with functional sensitivities of 0.2–0.3 ng/mL have a sensitivity and specificity of 54–63 % and 89 %, respectively, while assays with lower sensitivities of 0.02–0.1 ng/mL are associated with better sensitivity of 78–81 % but reduced specificity of 42–63 %.^18^ More recently, ultrasensitive assays were reported to give a sensitivity and specificity of 82.3% and 85.5–86.1 %, respectively.^17^ In our study, TgV to detect recurrence was shown to compare favorably with the above methods.

Furthermore, our findings are in principle concordant with the limited evidence available on Tg kinetics. Baudin et al.^19^ first confirmed the logical hypothesis that increasing Tg levels on repeated measurements indicated recurrence. This was a small study involving only 37 patients with Tg measured after thyroxine withdrawal. Another study verified the observation that rising Tg, with TSH either suppressed or stimulated, was associated with recurrence.^20^ In both of these studies, however, the rate of Tg rise above which recurrence could be reliably predicted was not reported. More recently, Miyauchi et al.^21^ showed that Tg doubling time less than one year significantly predicted locoregional recurrence and distant metastases. This study was, however, designed with excessively stringent inclusion criteria, excluding Tg samples taken with serum TSH ≥0.1 mIU/L. It should be noted that data are lacking regarding the appropriate level of serum TSH for evaluation of Tg change, and that TSH suppression to below 0.1 mIU/L in all patients is contrary to current guidelines which instead recommend a range from <0.1 to 0.1–0.5 mIU/L to normal depending on individual risk.^4^ Patients with anti-Tg antibodies, defined by using cutoff values set for Hashimoto thyroiditis, were also excluded; the diagnostic cutoff level for anti-Tg antibodies in Tg kinetic studies and whether these patients should be excluded in such studies are not clear. Moreover, the calculation of Tg doubling time in the study required at least 4 serial measurements, necessitating prolonged follow-up before prognosis could be determined. All these factors limited the clinical application of the study results. In our study, on the other hand, Tg samples with simultaneous TSH within or lower than the reference range, irrespective of anti-Tg antibody status, were included. TgV was calculated on the basis of 2–3 serial Tg measurements 6 months apart, allowing accurate recurrence prediction over a minimal time interval.

Regarding its prognostic ability, Tg value measured at a single time point did not correlate significantly with survival.^22^^,^ 23 However, the trend of Tg on serial measurements was shown to predict survival in the current and previous studies.^21^

TgV may therefore reflect disease aggressiveness. In patients with rising Tg but no other clinical evidence of disease, given that a TgV value above the cutoff predicted recurrence, especially metastases, its potential role to guide the administration of radioiodine or novel molecular targeted agents could be evaluated in future studies. On the other hand, among the patients with recurrent disease, TgV was below the proposed cutoff in 5 (17.2 %), including 3 (10.3 %) with a negative TgV signifying progressively declining Tg levels. This observation is reflected in the imperfect sensitivity and specificity associated with the cutoff, and it highlights the importance of supplementary clinical and radiological examinations in the follow-up of PTC patients.

The current study has a few limitations. First, the analyzed sample size was modest. The median follow-up time was also relatively short, considering the natural history of thyroid cancer, where recurrence can occur decades after treatment. The low recurrence rate in PTC renders patient recruitment difficult; we chose to exclude patients treated before the year 2003 in order to ensure a homogeneous study population where the same Tg and TSH assays and scans were used. Patients with undetectable Tg levels throughout follow-up were excluded from analysis, leading to potential selection bias in the complete evaluation of sensitivity and specificity profile of TgV. Moreover, patients with raised Tg were at times investigated with imaging earlier than specified in the follow-up protocol (Supplementary Table 1); this might result in earlier detection of recurrence compared to patients with undetectable Tg levels. However, these are less likely to be significant pitfalls in the current study because we analyzed only patients with raised Tg.

Our results are yet to be validated in a separate cohort. Whether clinical outcome can be improved when treatment decision is based on TgV has not been addressed. Finally, the role of TgV in patients with subtotal thyroidectomy, in those without radioiodine ablation, and in those with metastatic disease should be investigated in further studies.

In conclusion, TgV predicts recurrence with high sensitivity and specificity, and it is a prognosticator of survival in postthyroidectomy and postablation PTC patients with detectable Tg. Determination of TgV is simple and clinically applicable, while avoiding adverse effects and patient inconvenience associated with TSH stimulation. However, further studies are necessary to validate our results, and supplementary clinical and radiological examinations remain essential in the follow of up PTC patients.

## Electronic supplementary material

Below is the link to the electronic supplementary material.

Supplementary material 1 (DOC 28 kb)
