# Supplementary material for: Is There a Role for Unstimulated Thyroglobulin Velocity in Predicting Recurrence in Papillary Thyroid Carcinoma Patients with Detectable Thyroglobulin after Radioiodine Ablation?
Source: Ann Surg Oncol. 2012 May 11;19(11):3479–85. doi: 10.1245/s10434-012-2391-6 (PMC3442160; doi:10.1245/s10434-012-2391-6)
Supplement: Supplementary file 1 — Supplementary material 1 (DOC 28 kb) [file 10434_2012_2391_MOESM1_ESM.doc]

Supplementary Table 1. Imaging studies performed in addition to or earlier than those specified in the follow up protocol, as investigation for raised Tg levels.

| **Imaging** | **Recurrent patients (%)** | **Disease-free patients (%)** |
| --- | --- | --- |
| **WBS** | 9 (31.0) | 4 (6.9) |
| **PETCT** | 1 (3.4) | 3 (5.2) |
| **CT/ MRI** | 1 (3.4) | 0 (0.0) |
| **USG** | 2 (6.9) | 5 (8.6) |
| **Total no. of patients** | **29** | **58** |
